# Intriguing three-component four-layer stacking nanostructures formed by the self-assembly of main-chain liquid-crystalline polyurethane elastomers, as analyzed by two-dimensional small-angle X-ray scattering

**DOI:** 10.1039/d6ra01101b

**Published:** 2026-04-21

**Authors:** Yuki Hasegawa, Tomoka Noda, Seiji Iseki, Tatsuya Endo, Yume Sugino, Shinichi Sakurai

**Affiliations:** a Second Research Dept., Central Research Center R&D Headquarters, Toyo Tire Corporation, 3-10-1, Yato Kawanishi Hyogo 666-0131 Japan; b Department of Biobased Materials Science, Faculty of Fiber Science and Engineering, Kyoto Institute of Technology 1 Hashikami-cho, Sakyo-ku Kyoto 606-8585 Japan shin@kit.ac.jp

## Abstract

A peculiar two-dimensional small-angle X-ray scattering (2D-SAXS) pattern, exhibiting an X-shaped feature, was observed for a main-chain liquid-crystalline (LC) polymer in the uniaxially stretched state. The main-chain LC polymer examined in this study exhibits shape memory depending on temperature, which can be applied as a novel thermoresponsive soft actuator with a highly accurate and robust performance. The one-dimensional SAXS profile indicates a strange feature that is the unusually low intensity of the first-order reflection peak compared with the intensity of the second-order peak (inversion of the intensity). These peaks are ascribed to the periodic stacking of LC and other component layers. To explain this peculiar SAXS profile, model calculations were conducted by the Fourier transform of a plausible electron density profile. As a result, it was found that physical constraints in the main-chain LC polymer due to the forced formation of the LC phase by mesogenic units caused the extraordinarily sparse (low mass density) layer of the normal alkane moiety in a three-component four-layer stacking nanostructure. Other effects of physical constraints in the main-chain LC polymer on thermal properties were examined by differential scanning calorimetry (DSC) analysis. The DSC results reveal that the melting temperature (*T*_m_) of the LC phase and the degree of liquid crystallinity are lowered in the main-chain LC polymer compared with the precursory polyol biphenyl sample. The reflection peaks are broadened in the main-chain LC polymer, suggesting an extraordinary reduction in the grains of the stacking layers, to which the tremendous lowering of *T*_m_ of the LC phase is also ascribed. Thus, many effects due to physical constraints in the main-chain LC polymer were found. To further reveal the details of the nanostructure, the 2D-SAXS patterns exhibiting the X-shaped feature were measured during uniaxial elongation. Then, it was found that the crossing angle between the two representative directions in which the reflection peaks appeared increased and leveled off at the limiting value of 64°, which implies an inclination angle of 32° for the mesogenic units in the LC layer.

## Abstract 2

A peculiar small-angle X-ray scattering pattern, exhibiting an X-shaped feature, was observed for a main-chain liquid-crystalline polymer in the uniaxially stretched state.

## Graphical abstract

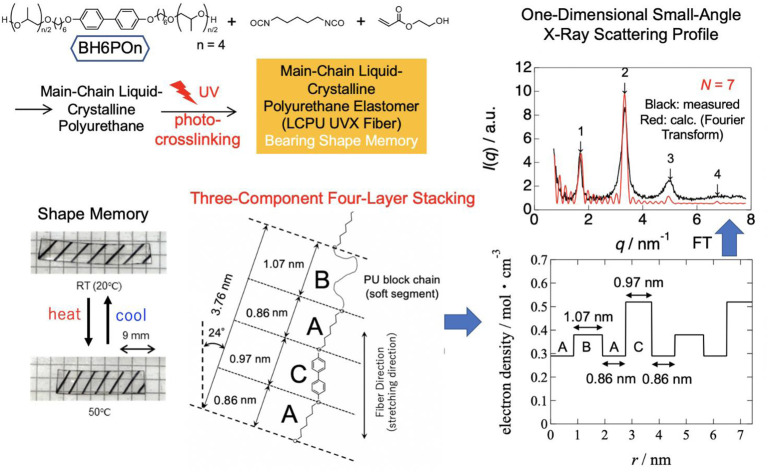

## Introduction

The development of soft materials with high performance and functionality has been one of the main targets in polymer materials science and technology.^1–8^ Among many strategies to achieve such developments, the utilization of thermoreversible melting and formation of liquid crystalline (LC) phase is notable.^9–29^ There are two representative categories of liquid crystalline polymers: side-chain and main-chain types.^30^ The former includes mesogenic units as side groups to the polymer chain, while the latter contains mesogenic units in the main chain of the polymer. The physical constraints on the formation of the LC phase for the mesogenic units are much stronger in the latter case than in the former case. Although such strong constraints suppress the formation of the LC phase, they may preserve the positional memory of the LC domains present before melting, enabling the material to retrieve almost the same original structure upon cooling below the melting temperature (*T*_m_). This idea can be significant to produce materials with a shape-memory effect depending on temperature. Thus, main-chain-type LC polymers are preferred for this purpose. Additionally, chemical crosslinking, introduced to impart elastomeric properties to main-chain LC polymer materials,^11,13,20,24,26,30^ strengthens the physical constraints. Considering the application of the material for soft actuators, such a shape-memory effect must be translated into stress and strain. This can be achieved by the uniaxial elongation of the material in its initial state. Then, structural transformations (the melting or formation of the LC phase) can be translated into changes in specimen dimensions. According to this idea, Hasegawa *et al.*^31^ developed a material that meets the requirement for the quick size changes in response to temperature variations. Since the analysis of the nanostructure of this material is important to understand the structural origin of such performance, we describe the details of the nanostructure of this material as analyzed by two-dimensional small- and wide-angle X-ray scattering (2D-SWAXS) measurements. By comparing the result with those of the precursory polyol specimen, the effects of strong physical constraints on the nanostructure of the main-chain LC elastomer (crosslinked LC polymer network) are discussed. To further analyze the detailed structure, the specimen was subjected to uniaxial stretching, and the two-dimensional small-angle X-ray scattering (2D-SAXS) patterns were measured as a function of the elongation ratio. Differential scanning calorimetry (DSC) measurements were also conducted to understand the effects on the thermal properties. Based on the DSC results, the degree of liquid crystallinity was evaluated to quantitatively assess the constraints on the formation of the LC phase imposed by the main-chain LC elastomer.

**Scheme 1:**
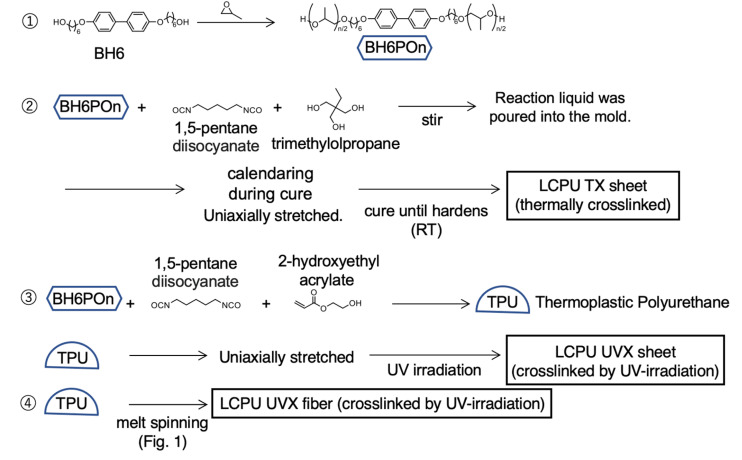
Synthetic routes and processing of the LCPU TX sheets, LCPU UVX sheets, and LCPU UVX fiber specimens.

According to the chemical structure of the main-chain LC polymer used in this study (Scheme 1), it is classified as a multiblock-type copolymer. Although the simplest chemical structure is a diblock copolymer comprising an LC and another moiety,^32,33^ this simplest structure does not cause the same level of physical constraint as a main-chain LC polymer, wherein both ends of the mesogenic unit are chemically bonded to amorphous moieties. In this regard, the physical constraint is comparatively intense in the case of an ABA-type triblock copolymer.^34–40^ Therefore, we summarize the nanostructural analysis, by 2D-SWAXS, of an ABA-type triblock copolymer where the component B is liquid-crystalline.

Although there are many studies reporting the results of 2D-SWAXS measurements for the case of the side-chain-type LC block copolymers,^41–47^ the number is limited for the literature reporting the case of main-chain LC polymers.^32–40^ Briefly summarizing the literature on triblock copolymers^32–40^ or oriented main-chain LC polymers subjected to shear or magnetic fields,^48–50^ several reflection peaks are observed in the SAXS region, highly aligned in the direction parallel to the fiber axis or the direction of the applied field, while the signals concerning the ordering and spacing of mesogenic units can be observed directly in the wide-angle X-ray scattering (WAXS) region. The reflection peaks in the SAXS region are ascribed to the nanostructure; therefore, the morphology, such as the lamellar structure of the smectic C (SmC) phase, can be analyzed, as well as quantitative determination of the repeating periodicity of the layers. Some studies have utilized model calculations based on the pseudo two-phase approximation to evaluate the scattering function.^34–36,39,40^ Note here that some peculiar experimental 1D-SAXS profiles have been reported, such as the disappearance of even-number higher-order reflection peaks^34–36^ or the third-order reflection peak,^35,40^ depending on the composition of the LC block component. However, the intensity of the first-order reflection peak is always the highest among all of the reflection peaks in these reports. Although we have observed a very peculiar 1D-SAXS profile in our previous publication on the main-chain LC elastomer sample,^31^ whereby the second-order reflection peak showed appreciably higher intensity than the first-order reflection peak, there is only one literature reporting the same feature.^39^ However, even in this literature, there is no clear explanation provided for this peculiar feature. There is another literature reporting the 2D-SAXS pattern (however, not showing the “X-shaped” pattern) with seemingly weaker first-order reflection peak than the second-order reflection peak, although any 1D-SAXS profile clearly showing such a feature was not presented in this literature.^42^ As for the “X-shaped” 2D-SAXS pattern, there is no report in the literature. Thus, in this current paper, we propose a plausible nanostructural model for the explanation of these peculiar SAXS results.

The Fourier transform of the electron density variation can give the scattering function (actually, the square of the structure amplitude is the scattering intensity). By comparing the electron density variation profiles that best produce the experimentally observed scattering profiles with the components of the main-chain LC polymer, the nanostructure and its features can be elucidated, which in turn enables an understanding of the effects of physical constraints in the main-chain LC multiblock copolymer on nanostructure formation. Actually, a three-component four-layer stacking model can reasonably explain the experimentally observed peculiar 1D-SAXS profile, and the layer comprising the normal alkane moiety neighboring the LC layer is suggested to have extraordinarily low mass density (due to the sparse existing of the normal alkane moiety in the layer), as a result of the physical constraints imposed by the forced formation of the LC layer by the mesogenic units.

## Experimental section

The materials were synthesized using our previously reported procedure,^31^ as shown in Scheme 1. Firstly, 4,4′-bis(6-hydroxy-1-hexyloxy)biphenyl (BH6) was synthesized according to the method described in the literature.^51^ Then, the LC polyol (BH6POn) was synthesized from BH6. The reagent BH6 was purified after its synthesis by washing with ethanol in the suspension state and then filtered, and further by washing with an ethanol/water (1/1 by volume) mixture in the suspension state and then filtered. This washing process was repeated three times and then dried at 80 °C under reduced pressure. The amount of remaining water was determined to be 0.28% by the Karl Fischer method. The reaction product of BH6 was found to be contaminated by the by-products of bisphenol reacted with one and three hexanol molecules. High-performance liquid chromatography with time-of-flight mass spectroscopy (HPLC-TOF/MS) was utilized to separate these components, and the purity of BH6 was found to be 98.9%.

BH6 (800 g, 2.07 mol) and KOH (11.97 g, 0.300 mol) were dissolved in toluene (2.17 kg), and the mixture was stirred under a nitrogen atmosphere at 120 °C for 15 min. To this solution, propylene oxide (619 g, 10.7 mol) was added dropwise. The mixture was stirred at 120 °C for 3 h and then cooled to 60 °C. Furthermore, oxalic acid (11.0 g, 0.122 mol) was added. The mixture was stirred for 0.5 h at 80 °C, and then the mixture was filtered. After solvent evaporation, the crude product was washed with water (1 L) and then dried *in vacuo* to obtain BH6POn (pale yellow paste, 930 g) (Scheme 1①). The obtained BH6POn with *n* = 4.2 (as determined based on the results from affinity liquid chromatography combined with mass spectroscopy measurements) was pasty at 23 °C but became an almost transparent liquid upon heating at 60 °C or above. The product BH6POn was characterized by ^13^C-NMR, and the results are provided in the SI to confirm its chemical structure, as shown in Scheme 1, although the final product contained an impurity (propylene glycol oligomers).

One of the key points in the material design of liquid-crystalline polyurethane (LCPU) samples is the reduction of the size of the LC domain to decrease the melting temperature (*T*_m_) of the LC phase.^35,37,39,40^ To this end, propylene oxide was reacted with the LC moiety. Changing the amount of propylene oxide reacted enabled control of the *T*_m_ of the LCPU elastomers.

As shown in Scheme 1②, the LCPU TX sheet specimen was synthesized using BH6POn as a starting material through a polycondensation reaction between BH6POn, isocyanate, and a trifunctional crosslinker without solvents. The reagent BH6POn was purified after the reaction of BH6 with propylene oxide by washing with water, and then dried *in vacuo*. The purity was determined by LC-TOF/MS to be 90%. The remaining 10% was propylene glycol oligomers (PG oligomers; the molecular weight was about 400), which was produced by the reaction of propylene oxide initiated by a trace of water molecules still remaining in the reactant even after evacuation. Note that the PG oligomers were not eliminated from the product and used in the reaction to obtain the LCPU. Therefore, some PG oligomers were incorporated into the main chains of the final product of LCPU.A mixture of 1,5-pentane diisocyanate (75 g) and trimethylolpropane (5.84 g) was stirred at 80 °C for 60 min. Then, this mixture (5.63 g) was combined with BH6POn (15 g) and tin(ii) 2-ethylhexanoate (8.08 mg), stirred for 30 s at 80 °C, and poured into a mold, which was maintained at 80 °C for 90 min to allow trimethylolpropane acting as a crosslinker to react with the LC polyol (BH6POn), and then cooled to 23 °C. When the reaction was partially proceeded, the material was uniaxially stretched to induce orientation of the mesogenic units. For this purpose, the specimen sheet was removed from the mold, stretched fourfold at 23 °C, and cured until hardening to complete the chain extension and the crosslinking reaction. This sample is referred to as the LCPU TX sheet.

On the contrary, the LCPU UVX sheet specimen was synthesized through a polycondensation reaction between BH6POn and diisocyanate without solvents and chemically crosslinked by the irradiation of ultraviolet (UV) light. Note here that 2-hydroxyethyl acrylate was used for the purpose of photo-crosslinking. A mixture of 1,5-pentane diisocyanate (31.38 g) and 2-hydroxyethyl acrylate (2.4 g) was stirred for 30 s at 80 °C and then poured into a mold containing BH6POn (120 g). The mold was heated and maintained at 80 °C for 90 min and then cooled to 23 °C to obtain TPU (thermoplastic polyurethane), as shown in Scheme 1③. The number- and weight-average molecular weights (*M*_n_ and *M*_w_, respectively) of TPU were determined by size-exclusion chromatography (40 °C, tetrahydrofuran used as an eluent), yielding *M*_n_ = 3.09 × 10^4^ and *M*_w_ = 6.35 × 10^4^ (polystyrene equivalent). These values give the number of the BH6POn repeats in the main chain, which are 39 and 81, respectively. Such a large number results from the chain-extension reaction between diisocyanate and the polyol BH6POn. Note here that both ends of the resultant polymer are terminated with the 2-hydroxyethyl acrylate, which has a photo-reactive vinyl group. The TPU sheet was cut into strips, stretched fourfold at 23 °C, and then photo-crosslinked by UV irradiation using an EYE mini GRANDAGE ECS-1511U (EYE GRAPHICS CO., LTD, Tokyo, Japan). The power of the UV irradiation device was 1.5 kW, the irradiation time was 0.19 s, and the illuminance was about 330 mW cm^−2^. Then, the sheet was thermally annealed at around 60 °C for several tens of seconds and cooled to 23 °C to obtain the LCPU UVX sheet specimen. Notably, a transparent material was required to enable homogeneous penetration of UV light throughout the specimen, ensuring homogeneous photo-crosslinking. The strategy to obtain a transparent specimen involved restricting the LC domain to a nanometer size, which was the same strategy employed to reduce *T*_m_. Thus, the preparation through UV irradiation (UVX) differs from one-pot crosslinking *via* thermally induced polycondensation with crosslinkers (TX) in that the crosslinking step (achieved through UV irradiation) is separated from the chain-extension step (achieved through the thermally induced polycondensation reaction of hydroxy and isocyanate groups forming the urethane bonds). Hopefully, complicated network structures (dangling chain ends, closed loops, and physical entanglement of network chains) could be avoided by separating these steps.

**Fig. 1:**
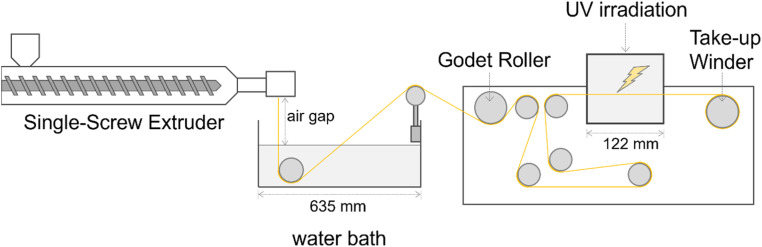
Schematic of the melt-span fiber processing of liquid-crystalline polyurethane (LCPU UVX fiber) with photo-crosslinking by UV irradiation.

An aliquot of TPU was melt extruded into fiber using a single-screw extruder (TECHNOVEL CORPORATION, Osaka, Japan) and immediately cooled in a cold water bath maintained at 20.5 °C (see the illustration of the setup in Fig. 1). The screw diameter of the single-screw extruder was 25 mm, and the length to diameter (*L*/*D*) ratio was 24. The rotational speed of the screw was 3 rpm. The temperature of the extruder and the spinning head was set to 80 °C. The molten polymer was forwarded to a spinning head having a spinneret with one hole of 2.0 mm in diameter. The extrusion temperature was kept at 80 °C. The distance from the spinneret to the surface of the water bath (air gap) was set to 25 cm. A godet roller was placed about 30 cm away from the water bath and rotated at a speed of 43.6 rpm. Before UV irradiation, the fiber was stretched to *λ*_X_ = 4, where *λ*_X_ denotes the stretching ratio as defined by the ratio of the rotation speeds of the rollers (before and after the stretching). The chemical crosslinking reaction was initiated by UV irradiation during the successive elongation process, just before winding the fiber onto a bobbin, producing the LCPU UVX fiber specimen. The power of the UV irradiation device was 1.5 kW, and the illuminance was about 160 mW cm^−2^.

For UV illumination, the difference between the sheet and fiber is due to the different processing conditions; for the sheet, the specimen was quiescently set in the container for UV illumination, while the fiber specimen continuously moved through the UV-irradiated region. To avoid physical interference in this case, the distance from the UV lamp to the fiber specimen should be maintained. Due to such conditions, the UV illuminance on the fiber specimen was lower than that on the sheet specimen. Swelling experiments were conducted in *N*,*N*-dimethylformamide (DMF) at room temperature to evaluate the crosslink density and gel fraction. Due to the widely distributed results of the degree of swelling in the range of 300–600%, it was not possible to estimate the crosslink density. However, it was found that the degree of swelling for the case of the fiber specimen was larger than that for the sheet specimen. Furthermore, the gel fraction of the former case was lower (70–84%) than that of the sheet specimen (85–94%). These results clearly indicate that the higher illuminance of UV results in a greater extent of crosslinking.

Take-up speed was set to 21.6 m min^−1^. The fineness of the fiber obtained was about 1280 dtex. Polarizing optical microscopy observations of the fabricated fiber revealed that the entire fiber appeared bright. On the contrary, when the fiber was rotated by 90°, the field of view became completely dark. These results suggest that the mesogenic units are uniaxially aligned parallel to the fiber axis.^31^

### Preparation of additional LCPU UVX sheet specimens

Furthermore, we prepared four additional types of TPU and LCPU UVX sheet specimens for DSC measurements: (A) press specimen, TPU pellet was melt pressed; (B) crosslinked Press specimen, the press specimen was chemically crosslinked by UV irradiation; (C) crosslinked uniaxial specimen, the press specimen was uniaxially stretched by 3.7-fold and chemically crosslinked by UV irradiation; and (D) LCE (liquid-crystalline elastomer) specimen, the crosslinked uniaxial specimen was once thermally annealed at temperature higher than *T*_m_ and then cooled to 23 °C.

### DSC measurements

DSC measurements were conducted on a DCS 214 Polyma (NETZSCH, Germany). The first-run measurement was conducted at a heating rate of 30 °C min^−1^ under a N_2_ purged condition. About 2 mg of the specimen was used for each measurement. According to DSC principles, a faster heating rate is preferred. However, thermal lag is of concern. To avoid such thermal lag, the amount of the sample used for DSC measurement should be kept as small as possible. Actually, we used ∼2 mg of sample while taking special care to have sufficient contact with the bottom of the aluminum pan. To verify whether this treatment is appropriate, we conducted additional DSC measurements at heating rates of 10 °C min^−1^ and 20 °C min^−1^. Fig. S4 shows the resulting DSC curves (first-run results), along with the peak temperatures and endothermic values as a function of the heating rate. As shown, the results for both are almost identical for heating rates of 20 °C min^−1^ and above. This indicates that the 10 °C min^−1^ was affected by the insensitive nature of the heating run of the DSC measurement so that the resulted value is not reliable. On the contrary, the results with the heating rate of 20 and 30 °C min^−1^ can be used as the ones free from the effect of the heating rate.

### 2D-SWAXS measurements

The 2D-SWAXS measurements were conducted using a D8 DISCOVER X-ray diffraction apparatus equipped with SAXS2 optics (Bruker AXS GmbH, Karlsruhe, Germany). The X-ray generator was operated at 50 kV and 1.0 mA to generate Cu Kα radiation (wavelength *λ* = 0.154 nm) with a microfocus beam (the beam diameter was 50 µm). A 2D X-ray mirror (MONTEL-P) and a collimator (UBC collimator) were used for the optical collimation of the X-ray beam. The distance between the specimen and the two-dimensional detector (VÅNTEC-500, Bruker AXS GmbH, Karlsruhe, Germany) was set at 300 mm for SAXS and 100 mm for SWAXS. The specimens were measured for 2500 s at 20 °C under a helium-gas purged condition. The temperature-dependent measurements of BH6POn were performed using a temperature control unit (TCPU-P, Bruker AXS GmbH, Karlsruhe, Germany) with an accuracy of ±0.2 °C. BH6POn was placed in a spacer with a thickness of 0.5 mm and sandwiched between polyimide films (Midfil NS, Kurabo Industries Ltd, Osaka, Japan) with a thickness of 50 µm. BH6POn was heated to 90 °C to confirm complete melting of the nanostructures (actually, no SWAXS peaks were observed).

## Results and discussion

**Fig. 2:**
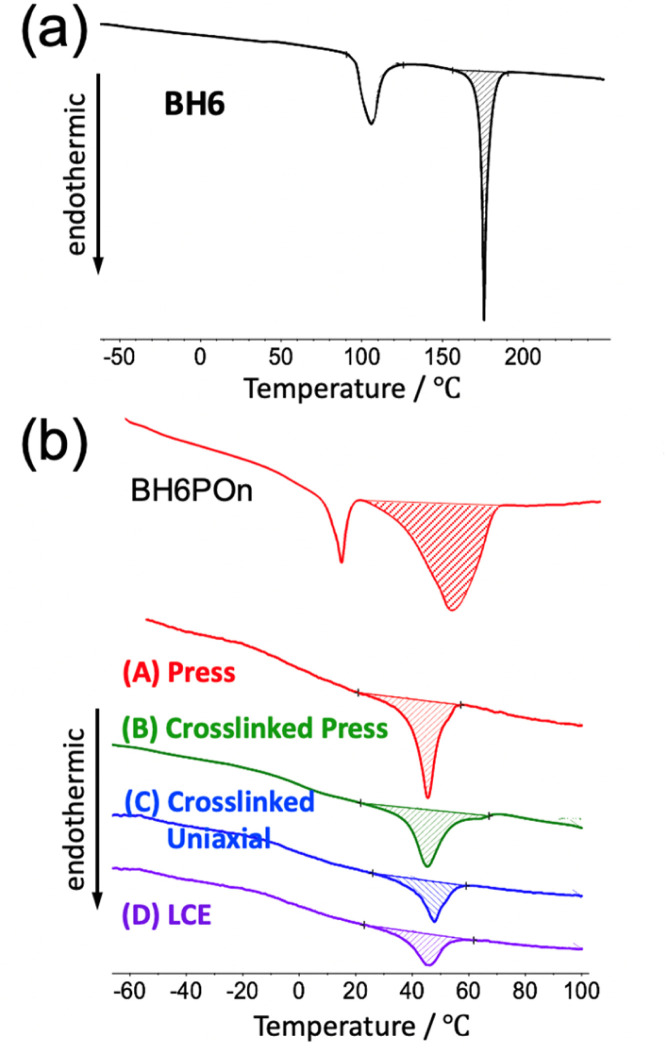
DSC curves for (a) BH6 and the (b) precursory polyol (BH6POn) and TPU and LCPU UVX sheet samples: (A) press, (B) crosslinked press, (C) crosslinked uniaxial, and (D) LCE (liquid-crystalline elastomer). The first-run measurements were conducted at a heating rate of 30 °C min^−1^.

Main-chain LC polymers can form a LC phase because of the presence of mesogenic units in the main chain of the polymer. However, both ends of the mesogenic unit are covalently bonded to the main chain so that the formation of the LC phase is expected to suffer constraints. Thus, it is further anticipated that not all mesogenic units participate in LC phase formation. To confirm this conjecture, the melting enthalpy of the LC phase (Δ*H*_m_) was measured by DSC, and the degree of liquid crystallinity (*χ*_LC_) was evaluated by taking the ratio of Δ*H*_m_ for the specimen and for the 100% liquid-crystalline phase. For this purpose, the biphenyl specimen (BH6) was measured as a reference sample. The results of the DSC measurements for BH6, the precursory polyol (BH6POn) and the liquid-crystalline polyurethane TPU and LCPU UVX sheet specimens (A)–(D) are shown in Fig. 2.

1 *χ*_LC_ = (Δ*H*_m,specimen_ × *x*_mes,BH6_)/(Δ*H*_m, BH6_ × *x*_mes,specimen_) × 100 (%),

where *x*_mes,BH6_ and *x*_mes,specimen_ denote the weight fraction of mesogenic units in the reference sample (BH6) and in the specimen, respectively. In the DSC curve for BH6, two endothermic peaks are observed. The lower-temperature peak (around 100 °C) is assigned to the crystal-to-LC transition, while the higher-temperature peak (around 175 °C) is assigned to the melting of the LC phase. Therefore, Δ*H*_m,BH6_ = 79.8 J g^−1^ was used for the evaluation of *χ*_LC_. Thus, evaluated *χ*_LC_ values are summarized in Table 1.

**Table 1:** Degree of liquid crystallinity of the precursory polyol (BH6POn) and liquid-crystalline polyurethane (TPU and LCPU UVX sheet samples): (A) press, (B) crosslinked press, (C) crosslinked uniaxial, and (D) LCE specimens. The degree of liquid crystallinity (*χ*_LC_) was evaluated based on the melting enthalpy of the liquid-crystalline phase obtained from DSC measurements (according to eqn (1))

Specimens	Degree of liquid crystallinity/%
BH6POn	69.5
(A) Press	52.1
(B) Crosslinked press	51.2
(C) Crosslinked uniaxial	31.3
(D) LCE	29.3

The DSC curve for BH6POn shows double endothermic peaks at 15 °C and 54 °C. These temperatures are very much depressed compared to the *T*_m_ of BH6, which suggests very small domains of the LC phase. Actually, as discussed later, the mesogenic units in BH6POn are confined within the LC layer with a thickness of about 1 nm, resulting in only microscopic LC phases and causing a tremendous decrease in *T*_m_. The *χ*_LC_ value of 69.5% for this specimen also indicates that physical constraints on the mesogenic units hinder the formation of the LC phase. For the TPU and LCPU specimens, the DSC curves show a single endothermic peak at around 46 °C. This temperature is very much depressed compared to that of BH6 and even lower than that of BH6POn, which suggests very small domains of the LC phase. Since the thickness of the LC layer is identical for BH6POn and these specimens, as discussed later, the depression of *T*_m_ may suggest that *T*_m_ depends not only on the layer thickness but also on the extent of the LC phase in the direction parallel to the layer. This conjecture will be confirmed later by structural analysis using 2D-SAXS measurements.

Lower values of *χ*_LC_ summarized in Table 1 for (A) press, (B) crosslinked press, (C) crosslinked uniaxial, and (D) LCE specimens clearly indicate that the physical constraint in the main-chain LC polymer hinder the formation of the LC phase. Based on these values, the following discussion can be made. The ∼52% liquid crystallinity for specimens (A) and (B), compared with ∼70% for the precursory polyol (BH6POn) before the chain-extension reaction, clearly indicates a decrease in the fraction of mesogenic units forming the LC phase due to the physical constraint of the main-chain LC polymer, while the chemical crosslinking reaction hardly decreases the liquid crystallinity, indicating that UV irradiation introduces chemical crosslinking without damaging the LC phase. On the contrary, the ∼30% liquid crystallinity observed for specimens (C) and (D) indicates the destructive role of uniaxial stretching in the LC phase. The destructive role of uniaxial stretching on the LC phase can be confirmed by tensile stress-strain (SS) tests. The results of cyclic SS tests for specimens C and D are shown in the SI (Fig. S2). During the first stage of tensile elongation, yielding is observed, and during the reversing process, the stress is much lower than that during the elongation process. Furthermore, a large amount of the strain remains at the end of the reversing process. This clearly indicates that some layered nanostructure is destroyed in the first stage of the elongation. However, the second cycle of the elongation and the reversing process exhibit an elastomeric behavior. Although the stress level in the second stage of the reversing process remains lower than that in the second-stage elongation process, subsequent cycles exhibit similar SS curves. This ensures the stable elastomeric mechanical properties of the specimen once strained.

The identical values of *χ*_LC_ for specimens (C) and (D) seem to suggest the thermoreversibility of LC phase formation of the specimen (C). However, the lower value of *χ*_LC_ for specimen (C) compared to those for specimens (A) or (B) is ascribed to the uniaxial stretching of the specimen, and it might therefore be expected that specimen (D) would retrieve the ability of LC phase formation upon thermal annealing, which should result in a *χ*_LC_ of 52%. However, as shown in Table 1, this conjecture is found to be wrong. Therefore, it is concluded that LC phase formation can be thermally reversible in specimen (C) due to chemical crosslinks, which is the main condition for the temperature-dependent shape-memory behavior of the LCE specimen.

The reason for such a lower degree of liquid crystallinity observed in specimens C and D (∼30%) is again due to the disruptive role of elongation during the preparation, and the reversible formation of the ordering of the mesogenic groups is due to incomplete randomization of the mesogenic groups upon melting of the LC phase. In other words, upon melting, the regular ordering of the mesogenic units is disrupted, but the mesogens keep their spatial positions similar to those previously in the LC phase. Complete recovery of the order of the mesogenic units is considered to be kinetically hindered by crosslinks. In this regard, it is considered that there is a phenomenological reversibility of the nanostructure formation within a practical time range (on the order of several minutes), although a thermodynamic equilibrium state is not attained. However, such phenomenological reversibility of the degree of the liquid crystallinity in specimen D is actually the key feature of its shape memory.

**Fig. 3:**
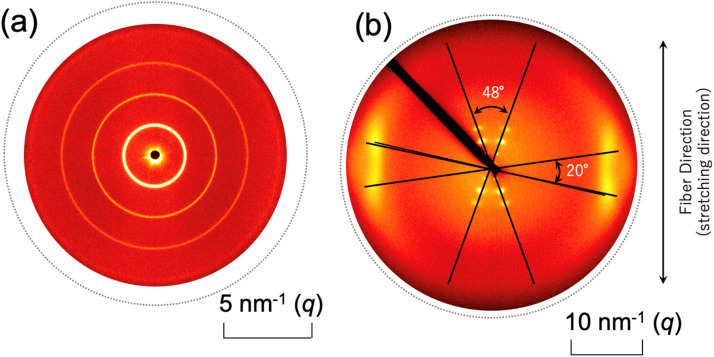
(a) Two-dimensional small-angle X-ray scattering (2D-SAXS) pattern of the precursory polyol (BH6POn) and (b) two-dimensional small- and wide-angle X-ray scattering (2D-SWAXS) pattern of the LCPU UVX fiber stretched to *λ*_X_ = 4 during its preparation process, measured at 23 °C.

**Fig. 4:**
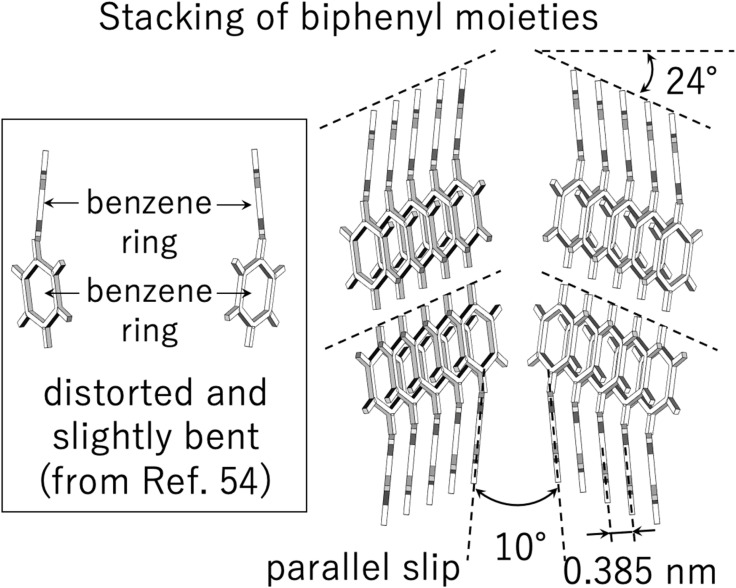
Schematic of the stacking of biphenyl moieties in the liquid-crystalline layer of the LCPU UVX fiber to explain the four spots (observed almost in the equatorial direction at *q* = 16.3 nm^−1^) in the 2D-SWAXS pattern shown in Fig. 3(b). Notably, the distorted and slightly bent structure of the biphenyl moiety presented here follows the conformation just prior to its melting, as proposed by Čapková *et al*.^54^ Then, the stacking of the distorted and slightly bent biphenyl moieties in the parallel slip manner is presented in four representative types. The spacing of 0.385 nm of the biphenyl moieties is evaluated from the peak position (*q* = 16.3 nm^−1^), which is slightly larger than the general value of the π–π stacking (0.32 nm). The inclination angle (24°) of the LC layer with respect to the fiber (stretching) direction (vertical direction) was determined from the 48° crossing angle between the two representative directions of the several peaks observed near the origin of the pattern (the “X-shaped” pattern) in the 2D-SWAXS pattern shown in Fig. 3(b).

The effects of physical constraints due to the main-chain LC polymer were thus confirmed by the reduction in *T*_m_ and *χ*_LC_. Now, their effects on nanostructure formation are examined by 2D-SAXS measurements. Fig. 3 shows a comparison of the 2D-SAXS patterns before and after chain extension: (a) the precursory polyol (BH6POn) and (b) the LCPU UVX fiber, measured at 23 °C. Here, *q* denotes the magnitude of the scattering vector, defined as *q* = (4π/*λ*)sin(*θ*/2), where *λ* and *θ* are the wavelength of the X-ray and the scattering angle, respectively. Since the polyol is not a polymer, it is not possible to stretch the specimen to orient the nanostructures. Thus, the isotropic ring peaks are observed. The appearance of these ring peaks clearly indicates the formation of stacked structures consisting of LC layers and layers comprising other components. On the other hand, the 2D-SWAXS pattern in Fig. 3(b) exhibits the orientation because the LCPU UVX fiber was stretched to *λ*_X_ = 4 during its preparation process. The elongation plays a role in orienting the polymer chains and the mesogenic units. Evidence of the orientation of the mesogenic units parallel to the fiber (stretching) direction is clearly observed as the peaks at the equatorial direction (perpendicular to the stretching direction) at *q* = 16.3 nm^−1^. From the peak position, the spacing between mesogenic units is evaluated as 0.385 nm, which is almost in accord with the general value of the π–π stacking (0.33–0.38 nm) of the biphenyl rings.^52,53^ However, a close examination of the 2D-WAXS pattern reveals that the peaks in the equatorial direction split into two spots at a slightly off equatorial direction. Although a similar four-spot pattern has been reported for biphenyl mesogens,^48,49^ the crossing angle of the diagonal directions (52°) is much larger than our result. Then, in this literature, the inclined orientation of the mesogenic units with respect to the stretching direction was proposed. On the contrary, we are sure that the parallel orientation of the biphenyl mesogens in our specimen because of the fourfold elongation applied during its preparation process. Therefore the observed four-spot 2D-WAXS pattern, with a 20° crossing angle between the diagonal lines connecting those two split spots on the left- and right-hand sides of the equatorial direction, is attributed to a slightly bent structure of the biphenyl rings, as schematically shown in Fig. 4 (see the biphenyl mesogen indicating that the benzene rings of the biphenyl mesogen are not placed on a common plane). Note that the distorted, slightly bent structure of the biphenyl moiety presented here follows the conformation just prior to the melting of the crystalline phase, as proposed by Čapková *et al*.^54^ Since our specimen has a *T*_m_ of 46 °C is close to the experimental temperature (23 °C), a distorted and slightly bent structure of the biphenyl moiety can be applied. Then, the stacking of the distorted and slightly bent biphenyl moieties in a parallel slipped manner is presented in four representative types. Based on this stacking of the benzene rings with a spacing of 0.385 nm, two spots corresponding to the model structures on the right-hand side appear in the second and fourth quadrants, while the other two spots corresponding to the model structures in the middle appear in the first and third quadrants. Thus, the crossing angle between the directions of these four spots should be 10°, slightly less than the observed one (20°). Note further that the inclination angle (24°) of the LC layer comprising the mesogenic units with respect to the fiber (stretching) direction (vertical direction) in Fig. 4 was determined from the 48° crossing angle between the two representative directions of the several peaks observed near the origin in the 2D-SAXS pattern (“X-shaped” pattern) shown in Fig. 3(b). In summary, the slightly bent biphenyl units stacked in a parallel slip manner (Fig. 4), which forms a smectic C phase, is thus proposed based on the experimental result of the 2D-SAXS pattern (the “X-shaped” pattern) shown in Fig. 3(b).

The slightly larger value of the π–π stacking distance (0.385 nm) compared with the literature range (0.33–0.38 nm) implies strong physical constraints on to the mesogenic groups in the LC layer, which is in good accord with the lower degree of liquid crystallinity (∼70%) observed for the BH6POn specimen (Table 1). In other words, although the mesogenic groups form the LC phase, they are forced to do so, and there would be trivial differences between mesogenic groups that participate in the LC phase and those that do not.

**Fig. 5:**
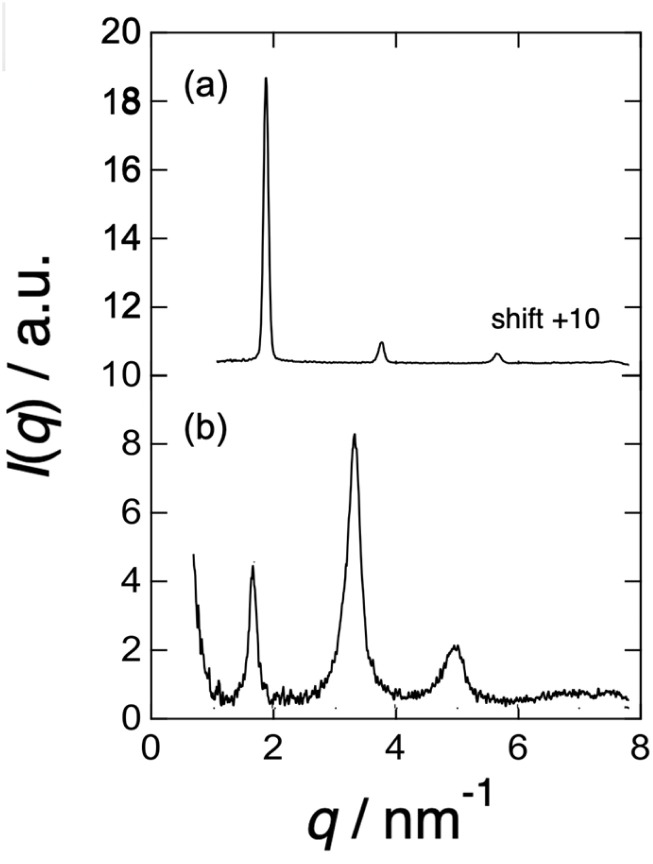
One-dimensional SAXS profiles for the (a) precursory polyol (BH6POn) and (b) LCPU UVX fiber, which was stretched to *λ*_X_ = 4 during the preparation process, measured at 23 °C. The former and latter ones are obtained by circular averaging of the 2D-SAXS pattern in Fig. 3(a) and sector averaging (along the directions of the several peaks observed) of the 2D-SWAXS pattern in Fig. 3(b), respectively. The profile for the polyol is shifted upward by +10 along the *y* axis to avoid overlap.

Fig. 5 shows the change in the one-dimensional SAXS (1D-SAXS) profiles before and after the chain extension reaction (from (a) to (b)). The profile in (a) for the precursory polyol (BH6POn) was obtained by circular averaging of the 2D-SAXS pattern shown in Fig. 3(a), while the profile in (b) for the LCPU UVX fiber was obtained by sector averaging (along the directions of the several peaks observed) of the 2D-SWAXS pattern in Fig. 3(b). First of all, the position (the *q* value) of the first-order peak differs between the two profiles. After chain extension, the repeat period of the layer stacking increases (from 3.35 nm to 3.76 nm). Second, the peaks become very broad after chain extension, suggesting a tremendous reduction in the size of the grains containing stacked LC layers. This results in the reduction in *T*_m_ (see Fig. 2 for the DSC curves). Finally, the relation of intensity of the first- and second-order peaks in profile (b) is unusual, whereby the first-order peak intensity is much lower than the second-order peak. In order to explain this strange profile, a model calculation of the SAXS profile is presented in the following paragraph.

**Fig. 6:**
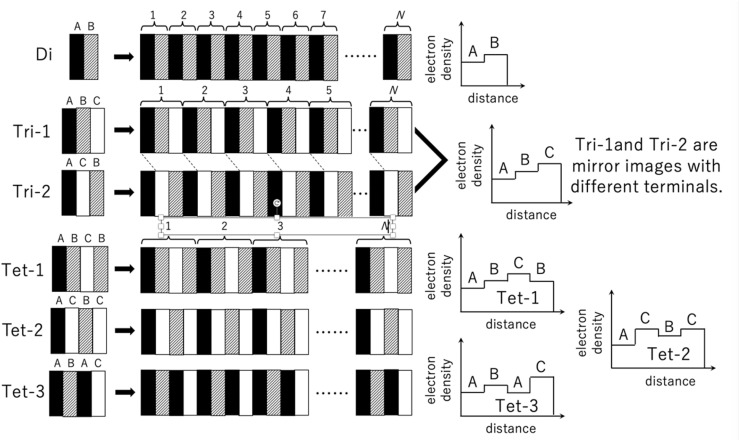
Schematic of the layered structures and the corresponding one-dimensional spatial variations in electron density *ρ*_e_ with A (black; the component having the lowest *ρ*_e_ = 0.3 mole electron cm^−3^), B (hatched; the component having the intermediate *ρ*_e_ = 0.4 mole electron cm^−3^), and C (white; the component having the highest *ρ*_e_ = 0.5 mole electron cm^−3^). The parameter *N* denotes the number of stacks used in the calculation of the 1D-SAXS profile. The layer thickness is identical for these layers.

**Fig. 7:**
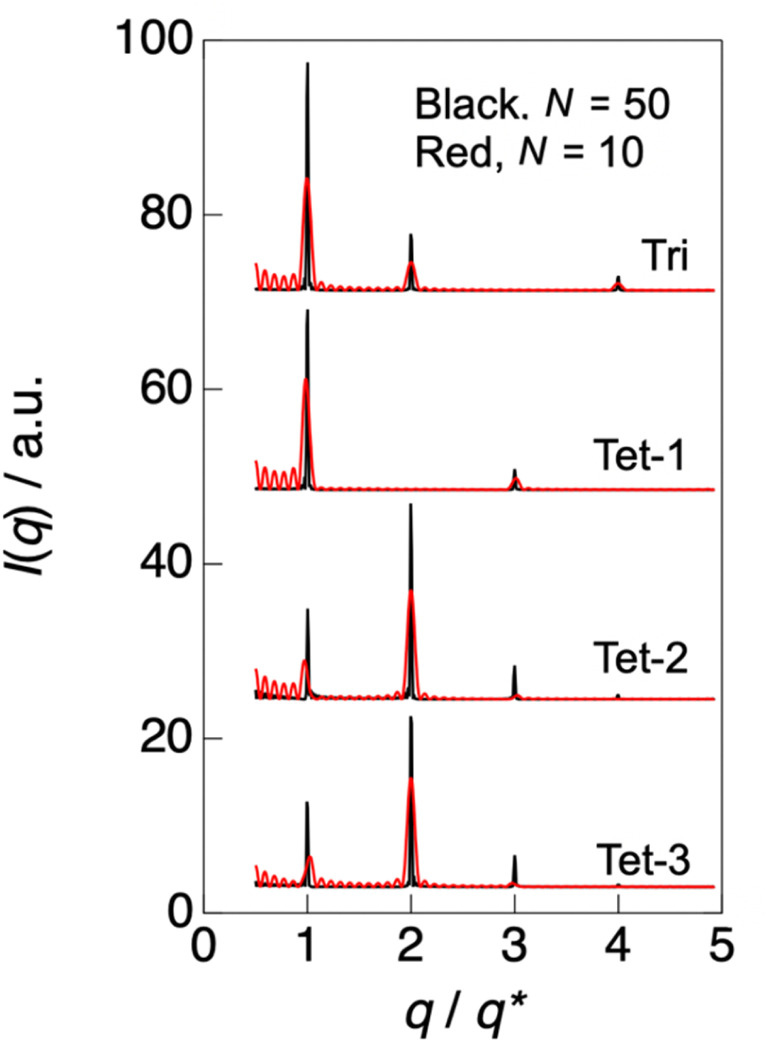
Calculated 1D-SAXS profiles using the variations in *ρ*_e_ shown in Fig. 6 for “Tri”, “Tet-1”, “Tet-2”, and “Tet-3”. Two curves are shown for each case, where the black and red lines are calculated using the number of stacks *N* = 50 and 10, respectively. The abscissa for *q* is normalized by the first-order peak position, q*.

The one-dimensional SAXS profile [*I*(*q*) *vs. q*] with the most intense second-order peak was calculated as the square magnitude (structure factor) of the Fourier transform (structure amplitude) of the one-dimensional spatial variation of the electron density *ρ*_e_. This automatically assumes structures of stacked layers. Since mesogenic groups in main-chain liquid-crystalline polymers have no other functionality than segregating into layers, the assumption of a layer-stacking structural model is reasonable. The number of stacks was appropriately changed to obtain the calculated SAXS profile (especially the peak broadening) as similar as possible to the measured SAXS profile. On the other hand, it should be noted that the lateral size of the layers was automatically assumed to be infinite. Getting started with the two-layer stacking case, as schematically shown in Fig. 6 as “Di,” the corresponding SAXS profile shows the most intense first-order peak among all of the reflection peaks, similar to the lamellar microdomains observed in the diblock copolymer case.^55^ Therefore, the “Di” model is not the case. Next, a more complicated structure is a three-layer stacking, as schematically shown in Fig. 6 as “Tri-1” or “Tri-2.” In this case, different electron density profiles are possible, where layer A has the lowest electron density value, and layers B and C have the intermediate and highest electron densities, respectively. However, “Tri-1” and “Tri-2” are intrinsically the same, as they are mirror images of each other when periodically repeated, as shown in Fig. 6. For these cases, the calculated SAXS profiles are shown in Fig. 7 using identical layer thicknesses for A, B, and C layers, with electron densities of 0.3, 0.4, and 0.5 mol electron/cm^3^, respectively. Note that the Fourier transform was performed using the software “Igor Pro 8” (WaveMetrics, Inc., Oregon, USA).^56^ The feature of the SAXS profile of the “Tri” case is the same as the “Di” case as the first-order peak is the highest and the higher-order peaks are lower than the first-order peak. Finally, we examined the case of the “four-layers stacking with three components A, B, and C. As schematically shown in Fig. 6, “Tet-1” (A/B/C/B), “Tet-2” (A/C/B/C), and “Tet-3” (A/B/A/C) can be considered as the different possibilities, where it should be noted that the electron density values of these components are the same as those used in the “Tri” case. As shown in Fig. 7, the calculated SAXS profile for “Tet-1” exhibits a similar feature, in which the first-order peak is the most intense. On the contrary, for both “Tet-2” and “Tet-3,” the SAXS profiles are almost identical to each other. This is because the symmetric aspect of scattering where the scattering function is invariant upon the exchange of the electron density of the highest one with the lowest one. For instance, in the case of “Di,” upon the exchange of A and B, the scattering function does not change. For the case of “Tri”, the exchange of A and C is relevant. As for the “Tet” case, the same exchange (A↔C) is also relevant. Therefore, the identical scattering functions observed for A/C/B/C and A/B/A/C can be understood from this symmetry. Upon such an exchange, A/B/A/C is transformed to C/B/C/A, and this is the mirror image of A/C/B/C. Thus, in principle, it seems to be impossible to distinguish A/C/B/C from A/B/A/C. However, when examining the effect of the number of stacks on the scattering function, a very interesting difference is observed. In Fig. 7, the black and red profiles are the results of setting the number of stacks *N* = 50 and 10, respectively. A decrease in this number results in the broadening of the peaks. Furthermore, not only peak broadening, peak shifting is observed, particularly for the first- and the third-order peaks. For the cases of “Tet-1” and “Tet-2,” the peaks shift towards lower and higher *q* directions, respectively. On the contrary, for the case of “Tet-3,” the peaks shift in the opposite direction. Thus, these different features can be applied to distinguish “Tet-2” (A/C/B/C) from “Tet-3” (A/B/A/C). Since the peak-shift behavior in the case of “Tet-3” is reasonable to obtain the SAXS profile similar to the measured one for the LCPU UVX fiber, the A/B/A/C type is chosen as the candidate for the final stage of the SAXS profile analysis.

**Fig. 8:**
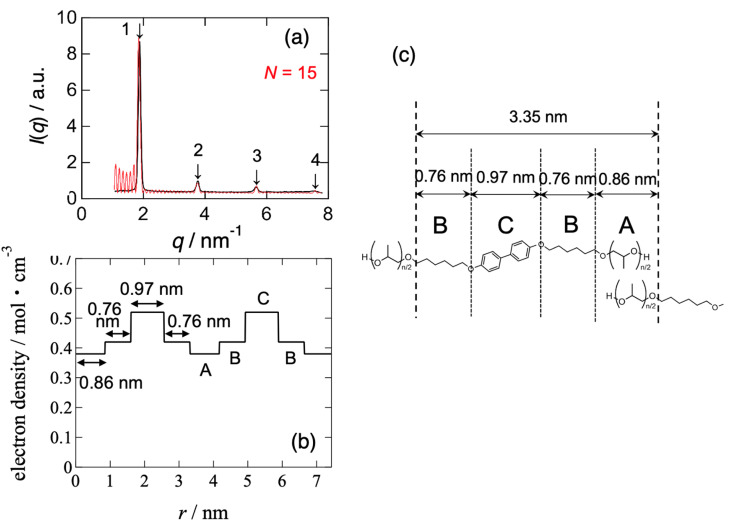
(a) SAXS profile (black curve) compared with the result of the Fourier transform (FT; the square of the structure amplitude) analysis, (b) electron density modulation used for the FT analysis, and (c) corresponding chemical structures of the layers for the precursory polyol (BH6POn).

**Fig. 9:**
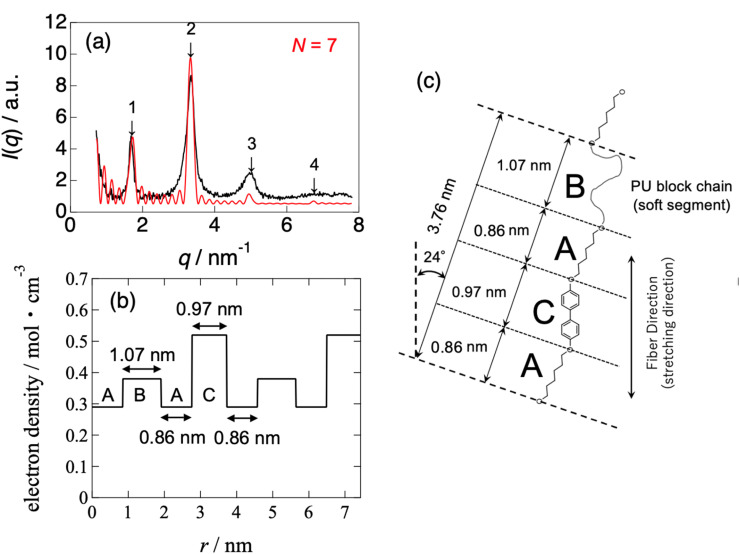
(a) SAXS profile (black curve) compared with the results of the Fourier transform (FT; the square of the structure amplitudes) analysis, (b) electron density modulation used for the FT analysis, and (c) corresponding chemical structures of the layers for the LCPU UVX fiber stretched at *λ*_X_ = 4 during the preparation process. The illustration in part (c) is inclined by 24° because of the inclination angle (24°) of the LC layer with respect to the fiber (stretching) direction (vertical direction), which was determined from the 48° crossing angle between the two representative directions of the “X-shaped” pattern observed in the 2D-SWAXS pattern shown in Fig. 3(b).

Fig. 8 and 9 show the best-calculated SAXS profiles for the BH6POn and the LCPU UVX fiber, respectively. It should be noted that the analysis of the BH6POn was performed to determine the layer thickness of A, B, and C with their values of *ρ*_e_. As the measured SAXS profile exhibits the most intense first-order peak, the “Tet-1” (A/B/C/B) model was chosen. As shown in Fig. 8, the calculated SAXS profile perfectly matches the measured one, which was obtained by setting a layer thickness of 0.86 nm for A (*ρ*_e_ of 0.38 mol electron cm^−3^), corresponding to the terminal propylene glycol moiety; 0.76 nm for B (*ρ*_e_ = 0.42 mol electron cm^−3^), corresponding to the normal alkane moiety (CH_2_)_6_ next to the LC layer; 0.97 nm for C (*ρ*_e_ = 0.52 mol electron cm^−3^) for the LC layer within the A/B/C/B model, and the number of stacks to *N* = 15. For the LCPU UVX fiber, the “Tet-3” (A/B/A/C) model was applied for the calculation of the best SAXS profile shown in Fig. 9, which was obtained by setting a layer thickness of 0.86 nm for A, corresponding to the normal alkane moiety (CH_2_)_6_ next to the LC layer; 1.07 nm for B, corresponding to the PU block chains; 0.97 nm for C, corresponding to the LC layer in the A/B/A/C model, and the number of stacks to *N* = 7. The values of *ρ*_e_ were appropriately changed to produce the SAXS profile similar to the measured one. The best agreement, as shown in Fig. 9, was obtained using *ρ*_e_ values of 0.29, 0.38, and 0.52 mol electrons cm^−3^ for the A, B, and C layers, respectively. Note here that the thickness and *ρ*_e_ of the LC layer used in the calculation were determined in advance by calculating the SAXS profile of BH6POn. As shown in Fig. 9, the first- and second-order peaks are well reproduced, and the calculated SAXS profile agrees well with the experimental data up to the fourth-order peak, although the agreement for the third-order peak is less accurate.

Since *N* stands for the number of repeating units in a grain, the grain size perpendicular to the LC layer can be estimated by the multiplication of the structural repeat periodicity (3.35 and 3.76 nm; note that these are not the layer thickness) by the number of stacks *N*, which is 15 and 7 for BH6POn and the LCPU UVX fiber (Fig. 8 and 9), respectively. Thus, the grain sizes are estimated to be 50.3 and 26.3 nm for BH6POn and the LCPU UVX fiber, respectively. The grain size can also be estimated from the width of the first-order reflection peak by the Scherrer equation, or more rigorously from the widths of both first- and higher-order reflection peaks by Hosemann's paracrystal theory.^57^ As shown in the SI, the grain size perpendicular to the LC layers was evaluated as 27.2 nm for the LCPU UVX fiber according to the reported method using Hosemann's theory. Thus, the validity of the SAXS calculation shown in the current study is confirmed. If the similarity of the grain shape is assumed, the decrease in the grain size upon the chain extension reaction suggests that the lateral size of the LC layer is considered to be decreased; therefore, the decrease in *T*_m_ (the DSC results) upon chain extension can be explained.

Here, the calculated curves (red) are closely compared with the observed profile (black). As shown in Fig. 8(a), not only the intensity but also the peak width are well reproduced up to the fourth-order peaks. On the other hand, the agreement is less satisfactory for the case shown in Fig. 9(a). For the latter case, we considered expressing the first-order peak intensity and its width as a first priority, and then expressing the second-order peak preferably. As a second priority, the width of the second-order peak near its maximum was reproduced while attempting to capture its width near the tail as much as possible. As a result, the third-order peak could not be reproduced by the calculation, as shown in Fig. 9(a). It is noted that matching between the black and red curves near the fourth-order peak is better. The reason for the poor agreement for the third-order peak may be ascribed to the oriented nanostructure of the specimen, as evidenced by the X-shaped SAXS pattern. However, the most favorable SAXS pattern for structural analysis is a homogeneous powder pattern obtained from an unoriented nanostructure. We are currently conducting nanostructure analyses to achieve a more reliable and reasonable understanding of the nanostructures. Finally, it should be noted that in both cases in Fig. 8(a) and 9(a), there are many noisy peaks in the *q* range lower than the *q* of the first-order peak. These disappear completely when the number of repeating units *N* is increased. Therefore, this may be due to the one-dimensional Fourier transform calculation with a finite size of the grain, in which the contribution from the ends of the electron density variation (cutting-off of the variation) in the grain is comparatively large, as observed in the calculation shown in Fig. 8(a) and 9(a). Although the grain size has a distribution in a real sample, the SAXS model calculation is limited to the mono-disperse case, which produces such noisy peaks. It may also be ascribed to other limitations of the calculation of the SAXS profile, including the assumption of infinite lateral size of the layers.

The chemical structures of the corresponding layers used for the calculation of the red curves in Fig. 8(a) and 9(a) are shown in Fig. 8(c) and 9(c), respectively. These are typical of “Three-Component Four-Layer Stacking” nanostructures due to the formation of the LC layers. The SAXS profile shown in Fig. 8(a) is explained more easily by assuming a terrace-shaped variation in the electron density, in which the LC layer bears the highest electron density of 0.52 mol electrons/cm^3^, estimated from a mass density value of 1.04 g cm^−3^ (ref. 58) Then, the layer composed of the normal alkane moiety next to the LC layer exhibits an intermediate electron density of 0.42 mol electrons cm^−3^, with the slightly lower value for the layer comprising the propylene glycol moiety with a hydroxyl terminal (0.38 mol electrons cm^−3^). On the other hand, to explain the strange SAXS profile shown in Fig. 9(a), a three-component four-layer stacking with an electron density variation following a trench-and-bump shape, as shown in Fig. 9(c), is used, where layer C is the LC phase, having an electron density of 0.52 mol electron cm^−3^, which is set to be identical to the one used in Fig. 8(b). On the contrary, the electron density of the normal alkane moiety-containing layer is unusually low (decreased from 0.42 mol electrons cm^−3^ in BH6POn to 0.29 mol electron cm^−3^ in LCPU (31% reduction)). This situation is induced by the forced formation of the LC phase, where mesogenic units are aligned, causing physical constraints on the normal alkane moiety to form its layer with very low mass density. Note here that both ends of the normal alkane moiety are covalently bonded to the mesogenic unit and the urethane moiety in the main chain of the polymer, which easily imposes the physical constraint on the normal alkane moiety, causing an appreciable increase in the layer thickness (from 0.76 to 0.86 nm). Such a feature of the main-chain LC polymer, in which layer A is forced to form with an extraordinarily low electron density, results in the strange SAXS profile shown in Fig. 9(a). On the other hand, the thickening of the polyurethane layer (from 0.86 to 1.07 nm) upon chain extension is ascribed to the increased volume by the addition of 1,5-pentane diisocyanate and 2-hydroxyethyl acrylate to the hydroxyl terminal of the precursory polyol (BH6POn). Note that the same values of *ρ*_e_ (0.38 mol electrons cm^−3^) were used for these layers in the calculations, as shown in Fig. 8(b) and 9(b).

**Fig. 10:**
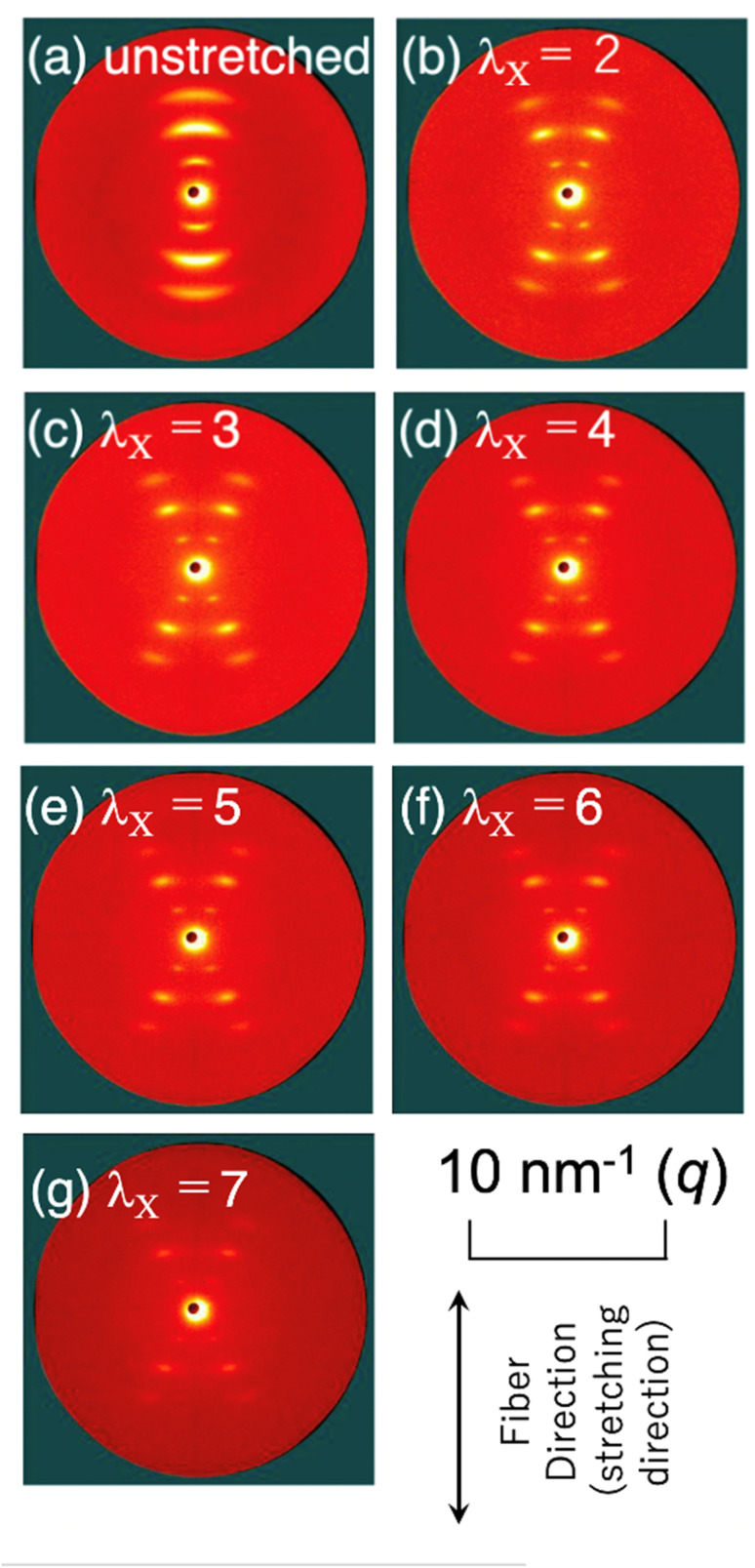
Changes in the 2D-SAXS patterns along with the elongation of the LCPU UVX fiber (a) at unstretched state, and (b–g) at the stretching ratio of 2, 3, 4, 5, 6 and 7, where *λ*_X_ indicates the elongation ratio, measured at 23 °C.

To further reveal the details of the nanostructure, changes in the 2D-SAXS exhibiting the X-shaped pattern were measured during uniaxial elongation. Fig. 10 shows the changes in the 2D-SAXS patterns upon the uniaxial stretching of the LCPU UVX fiber. The fiber and stretching directions are vertical. Note here that the fiber (before stretching) was previously heated and maintained around 60 °C for 10–30 seconds and cooled to 23 °C. In the original state (Fig. 10(a)), an oriented SAXS pattern was observed, with reflection peaks preferentially aligned in the direction parallel to the fiber direction, suggesting that the stacking layers are preferentially oriented with their stacking direction parallel to the fiber axis.

The purpose of stretching during the preparation of the fiber specimen is to induce orientation of the nanostructure (as shown in Fig. 10(a)). Additionally, the polymer network prepared by the photo-crosslinking (UV irradiation) is deformed upon stretching during preparation. Therefore, upon heating the specimen to a temperature above *T*_m_, the LC phase melts, which results in the relaxation of the deformed polymer network causing shrinkage of the specimen (see Fig. S3a in the SI). On the contrary, upon cooling, the LC phase reforms, and the original nanostructure is retrieved, resulting in the deformation of the polymer network and causing the specimen to stretch. This is the origin of the shape memory of the specimen. Therefore, it can be stated that the stretching of the specimen during preparation is the key to enabling temperature-induced shape memory. On the other hand, the purpose of the stretching experiment shown in Fig. 10 was to further develop the nanostructure analyses of the LC layer. Actually, with slight stretching, a characteristic X-shaped SAXS pattern appears, as shown in Fig. 10(b)–(g). Based on this feature, the inclination angle of the LC layer can be evaluated, as discussed in the following paragraph.

**Fig. 11:**
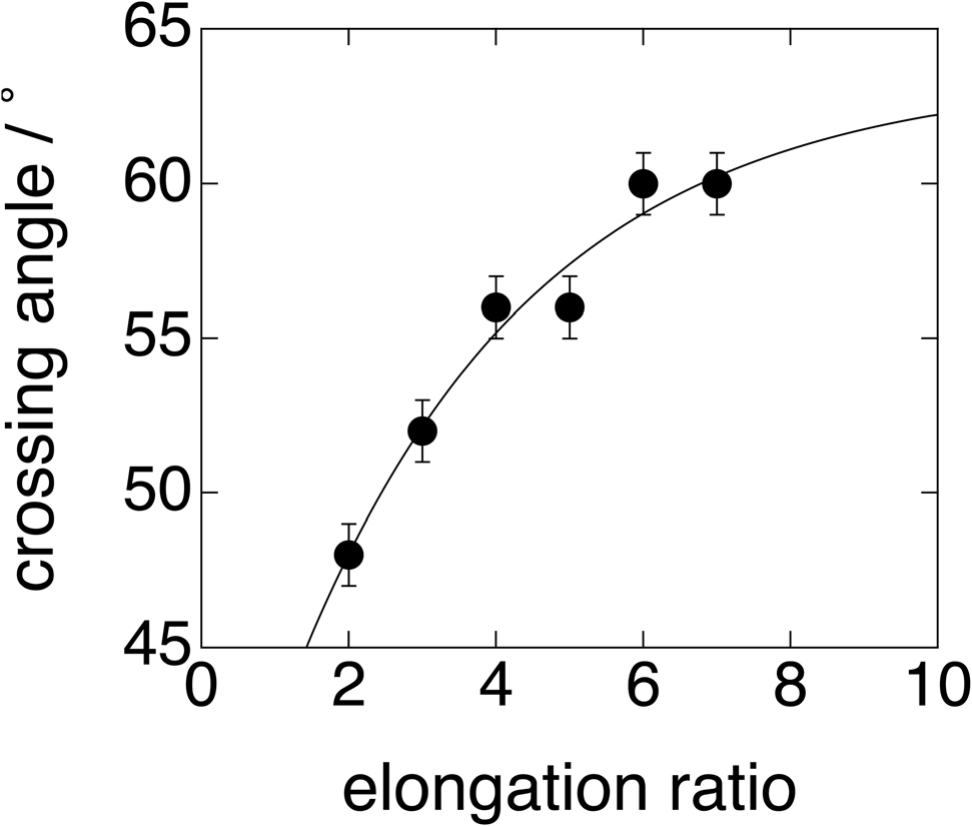
Plot of the crossing angle of the “X-shaped” 2D-SAXS pattern as a function of the elongation ratio, extracted from the results shown in Fig. 10.

Note that the X-shaped pattern (as shown in Fig. 3(b) for the fiber without thermal annealing at 60 °C for 10–30 seconds) transforms into the pattern shown in Fig. 10(a) after thermal annealing. This fact means that, upon melting of the LC phase, the uniaxially oriented polymer chains become somewhat relaxed, so that the layered structures reformed during cooling after thermal annealing are reorganized with the stacking direction of the LC layers aligned perpendicular to the fiber axis. Thus, an orientation in which the LC layers are perpendicular to the fiber axis is preferred as a thermodynamically stable state, which suggests a grain orientation with its long axis parallel to the fiber axis.^53^ Upon uniaxial elongation, the 2D-SAXS pattern changes into the X-shaped pattern shown in Fig. 10(b). This clearly indicates the parallel orientation of the mesogenic units in the elongation direction, resulting in the inclined orientation of the LC layers with respect to the elongation direction, as illustrated in Fig. 4 and 9(c), and retrieving the X-shaped 2D-SAXS pattern. It seems that almost no change is discernible for further elongation, although the peak intensity became lower with increasing elongation ratio. As a matter of fact, the position of the first-order peak remains unchanged even upon uniaxial elongation towards *λ*_X_ = 7 (Fig. 10(g)). The invariance of the repeating period and the decrease in peak intensity with increasing elongation may suggest no deformation of the layered structure in some grains, but the instantaneous fracture and disappearance of other grains upon elongation. This corresponds to the clear yielding observed in the stress-strain behavior at 23 °C (see the SI).^31^ The observed 2D-SAXS patterns reflect only the undeformed layered structures in the remaining intact grains of the stretched fiber. To closely examine the change in the crossing angle between the two representative directions where multiple diffraction peaks appear, the angle was evaluated from the 2D-SAXS patterns, and its change is plotted in Fig. 11 as a function of elongation ratio. Then, it is clearly found that the crossing angle appreciably increases with increasing elongation ratio. The increasing tendency can be fit by an exponential function, where its increasing tendency levels off around *λ*_X_ = 10, with a limiting crossing angle of 64°. This result indicates that the inclined orientation of the LC layers develops gradually along with elongation. Its limiting value (64°) implies an inclination angle of 32° for the mesogenic units in the LC layer.

It should be noted that there is no relation between the crossing angle and the bent structure of the biphenyl moiety, and they are independent of each other. The crossing angle is rather rationalized by the inclination of the biphenyl groups in the LC phase. The limiting value of 64° corresponds to twice the inclination angle by assuming a perfect orientation of the polymer chains (*i.e.*, mesogenic groups) parallel to the stretching direction. According to the literature,^54^ the stacking of the biphenyl groups occurs in a parallel, slipped manner to set a benzene ring at the middle of the C–C single bond connecting the two benzene rings within a biphenyl unit. In other words, the two benzene rings in a biphenyl unit are not completely stacked parallel to each other, and they are parallel slipped. Based on this condition and using the experimentally determined π–π stacking distance (0.385 nm), exactly the same inclination angle of 32° is obtained. Thus, the limiting crossing angle of 64° is found to be quite reasonable.

## Conclusions

The effects of the physical constraint of the main-chain LC polymer on thermal properties and nanostructures were examined by DSC and 2D-SWAXS measurements. Note that the main-chain liquid-crystalline polymer examined in this study exhibits a thermoresponsive shape memory. Namely, the length of the sheet specimen becomes longer below *T*_m_ and shorter above *T*_m_ of the LC phase in the specimen (*T*_m_ = 46 °C). Such a shrinking and expanding of the length of the specimen can be repeat up to 47 000 times by changing the temperature between 20 °C and 50 °C. The span of the length is macroscopic (7 mm), and the reproducibility of the size is very accurate (27 mm at 20 °C and 20 mm at 50 °C). Thus, the specimen can be applied as a novel thermoresponsive soft actuator with highly accurate and robust performance. The DSC results revealed that *T*_m_ of the LC phase and the degree of the liquid crystallinity are lowered in the main-chain LC polymer compared to the case of the precursory polyol biphenyl sample. The 2D-SAXS pattern of this polymer exhibits a very peculiar feature that is the X-shaped pattern, along with an unusually low intensity of the first-order reflection peak (compared to the intensity of the second-order peak). These peaks are ascribed to the repeating periodicity of the LC and other component layers. To explain this peculiar SAXS profile, model calculations were conducted by the Fourier transform (FT) of a plausible electron density variation, since X-ray elastic scattering can be rationalized by the FT of the electron density variations. As a result, it was found that the physical constraint of the main-chain LC polymer caused an extraordinarily sparse (low mass-density) layer of the normal alkane moiety (the A layer) in the three-component four-layer stacking (A/B/C/B) nanostructure, where layer C comprises the LC phase. Furthermore, the reflection peaks are broadened in the main-chain LC polymer, suggesting an extraordinary reduction in the grain size of the stacked layers. Thus, many effects due to the physical constraint of the main-chain LC polymer were found. To further reveal the details of the nanostructure, changes in the 2D-SAXS patterns exhibiting the X-shaped feature were measured under uniaxial elongation. Then, it was found that the crossing angle of the “X-shaped” SAXS pattern was increased, and the limiting value was found to be 64°, implying an inclination angle of 32° for the mesogenic units in the LC layer.

## Conflicts of interest

There are no conflicts of interest to declare.

## Supplementary Material

RA-016-D6RA01101B-s001

## Data Availability

Data are available from the corresponding author upon reasonable request. Supplementary information (SI): 1. Characterization of the product; 2. Mechanical property; 2.1. Stress–strain behavior; 2.2. Performance of the shape memory upon change in temperature; 3 Thermal property by DSC measurements; 3.1. Effects of the heating rate; 3.2. Difference of the DSC curves in the first-run of heating and that in the second-run of heating; 4. SAXS analysis of the grain size and contains the following figures; Fig. S1: ^13^C-NMR spectrum for the BH6POn. Fig. S2: results of the cycle test of stress–strain behavior at 23 °C for samples C and D. Fig. S3: shape memory (a) and results of heating and cooling cycle test between 20 °C and 50 °C of the LCPU specimen. Photograph (b) and changes in the length of the specimen (c) during the cycle test. Fig. S4: (a) comparison of the DSC curves in the first-run heating process of sample A with the heating rates of 10, 20, and 30 °C min^−1^. (b) Plots of *T*_m_ and Δ*H*_m_ as a function of the heating rate. Fig. S5: comparison of the results of the heating DSC curves (with the heating rate of 20 or 30 °C min^−1^) for the first run and second run for (a) samples A–D and (b) BH6 and BH6POn. Fig. S6: Hosemann plot by taking the square of Δ*q* as a function of the fourth-power of *m* (the order of the peak as *m* = 1, 2, 3, 4,…). See DOI: https://doi.org/10.1039/d6ra01101b.
