# Supplementary material for: Intriguing three-component four-layer stacking nanostructures formed by the self-assembly of main-chain liquid-crystalline polyurethane elastomers, as analyzed by two-dimensional small-angle X-ray scattering
Source: RSC Adv. 2026 Apr 21;16(23):20840–54. doi: 10.1039/d6ra01101b (PMC13097005; doi:10.1039/d6ra01101b)
Supplement: RA-016-D6RA01101B-s001 [file RA-016-D6RA01101B-s001.pdf]

Supplementary Information (SI) for RSC Advances.
This journal is © The Royal Society of Chemistry 2026

Supporting Information

Intriguing Three-Component Four-Layer Stacking Nanostructures Formed by Self-Assembly of Main-Chain Liquid-Crystalline Polyurethane Elastomers as Analyzed by Two-Dimensional Small-Angle X-Ray Scattering Technique

Yuki Hasegawa[1], Tomoka Noda[1], Seiji Iseki[1], Tatsuya Endo[1], Yume Sugino[2], Shinichi Sakurai[2]

[1]Second Research Dept., Central Research Center, R&D Headquarters, Toyo Tire Corporation, 3-10-1, Yato, Kawanishi, Hyogo 666-0131, Japan
[2]Department of Biobased Materials Science, Faculty of Fiber Science and Engineering, Kyoto Institute of Technology, 1 Hashikami-cho, Sakyo-ku, Kyoto 606-8585, Japan

KEYWORDS: main-chain liquid-crystalline polymer, photo crosslinking, elastomer, two-dimensional small- and wide-angle X-ray scattering, X-shaped pattern, three-component four-layer stacking, peculiar SAXS profile, differential scanning calorimetry, degree of liquid-crystallinity

1. Characterization of the product

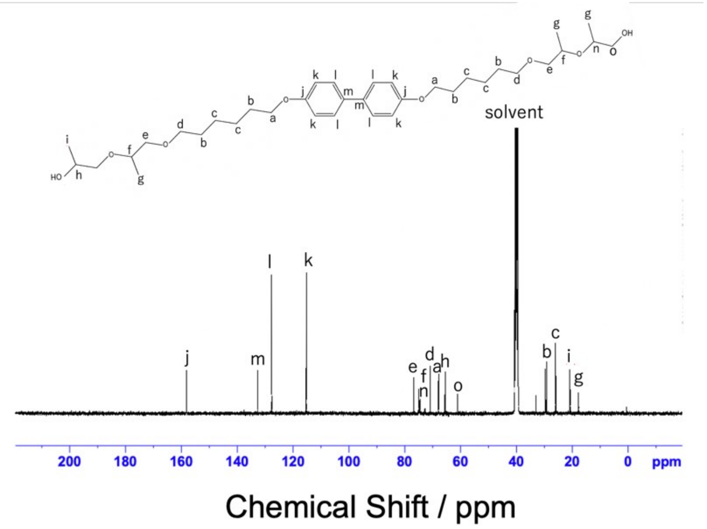

Figure S1. ^{13}C-NMR spectrum for the BH6POn.

The ^{13}C-NMR spectrum for the BH6POn is shown in Figure S1 with peak assignments of the chemical structure of the BH6POn, which was obtained by the reaction of BH6 and propylene oxide. Due to the difference of the head-to-tail or head-to-head

configurations, different chemical structures are available, which are illustrated in the chemical structure in Figure S1, as the former and the latter ones are, respectively, in the left-hand and the right-hand sides with respect to the central biphenyl group. Thus, all the peaks can be assigned and the expected product is confirmed.

2. Mechanical Property

1-1. Stress-strain behavior

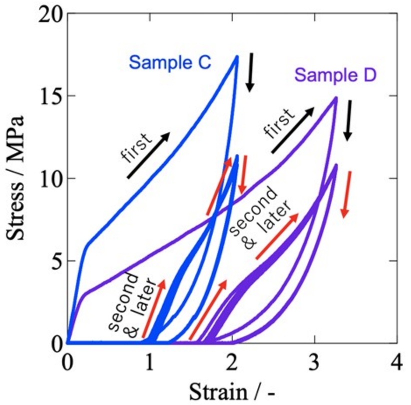

Figure S2. Results of the cycle test of stress–strain behavior at 23°C for Samples C and D.

1-2. Performance of the shape memory upon change in temperature

As shown in Figure S3a, the LCPU sample exhibits spontaneous length change upon heating and cooling (at 20 and 50˚C) as a shape memory effect. To further examine its robustness, heating and cooling cycle test between 20°C and 50°C was conducted under the mechanical load (105g or 307g) as shown in Figure S3b (phot), and the results shown in Figure S3c. This experiment was intended to demonstrate the long term durability of the macroscopic actuation behavior under constant load over tens of thousands of thermal cycles.

The cross-sectional dimensions of the specimen were 1.34 mm in thickness and 6.93 mm in width for the 107 kPa condition, and 1.43 mm in thickness and 7.02 mm in width for the 300 kPa condition. The total mass of the fixtures and weights attached to the specimen was 105.23 g and 307.3 g, corresponding to applied stresses of 107 kPa and 300 kPa, respectively. Immediately after attaching the fixtures, the initial specimen length (distance between the upper and lower fixtures) was measured to be 22 mm for both conditions. The specimen together with the fixtures was placed in a temperature-controlled chamber (PR-1J; ESPEC CORP., Osaka, Japan). The chamber atmosphere was not purged with nitrogen and remained under ambient air. Temperature

was controlled programmatically by repeatedly heating to 50°C and cooling to 20°C. After reaching each target temperature, the specimen was held at that temperature for 30 min before the subsequent heating or cooling step. The heating and cooling rates were not specifically accurately controlled.

The specimen length at 50°C was measured rapidly using a ruler, followed by measurement at 20°C after cooling. The specimen length was read using a scale with 1 mm increments, with an estimated reading error of ±0.5 mm. In this experiment, air cooling was employed, which is less efficient than the water-cooling conditions used in Figure 6; therefore, rapid changes in specimen length during the measurement are considered unlikely. The test was continued for approximately 3.5 years; however, the experiment was terminated after 47,000 cycles due to a failure of the temperature-controlled chamber.

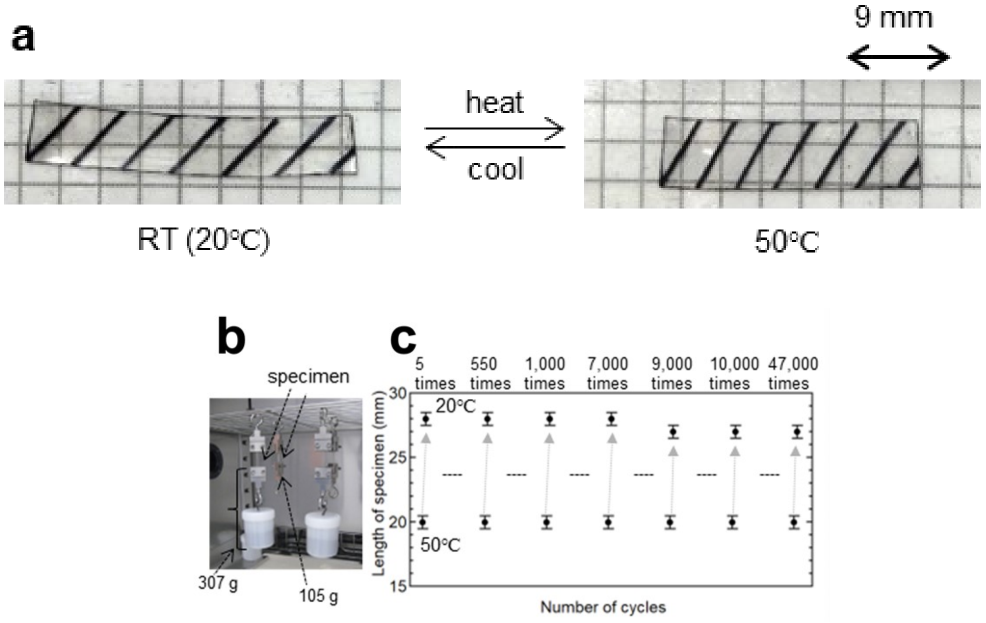

Figure S3. Shape memory (a) and results of heating and cooling cycle test between 20°C and 50°C of the LCPU specimen. Photograph (b) and changes in the length of the specimen (c) during the cycle test.

3. Thermal Property by DSC measurements

2-1. Effects of the heating rate

According to the DSC mechanism, the faster heating rate is preferred. To avoid thermal lag, the amount of the sample used for the DSC measurement should be as small as possible. Actually, we used ca. 2 mg with taking special care to have sufficiently large contact to the bottom of the alminum pan. To check whether this treatment is

appropriate, we conducted the additional DSC measurements with 10, 20, and 30˚C/min of the heating rates. Fig. S4 shows the resulted DSC curves (the first-run results) and the peak top temperature (T_m) and endotherms (ΔH_m) as a function of the heating rate. As shown, the results for the both are almost identical for the heating rate higher than 20˚C/min. This ensures the sufficient thermal contact and the 10˚C/min. was found to be affected by the insensitive nature of the heating run of the DSC measurement so that the resulted value is not reliable.

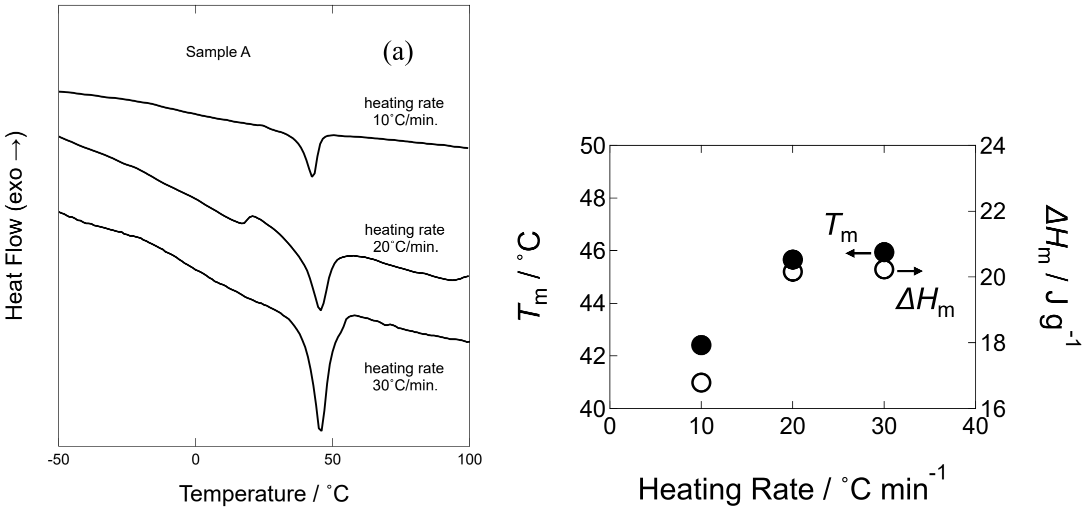

Figure S4. (a) Comparison of the DSC curves in the first-run heating process of sample A with the heating rates of 10, 20, and 30˚C/min. (b) Plots of T_m and ΔH_m as a function of the heating rate.

2-2. Difference of the DSC curves in the first-run of heating and that in the second-run of heating

Comparison of the results of the heating DSC curves (with the heating rate of 20 or 30 ˚C/min) for the first run and second run in Fig. S5a. For all of the samples A-D, it was observed in the second-run heating DSC curves that the peak temperature and the area of the endothermic peak (corresponding to the melting of the LC phase) shifted towards lower temperature and decreased, respectively. This is because the reformation of the LC layers during the cooling (about 33 min. with 10˚C/min. cooling rate) from the completely molten state was incomplete. Since it takes longer time, the result of the first-heating run is reliable for the analysis of the nanostructure formation. Thus, we will discuss in the main text based on the results of the first-heating run DSC curves. As for the reformation of the liquid-crystalline phase in the BH6 sample (Figure S5b), it is almost thermoreversible within the cooling process (about 33 min. with 10˚C/min. cooling rate) from the highest temperature of the end

of the first-run of the DSC down to the starting temperature of the second-run since the second-run of the heating DSC curve shown in Fig. S5b indicates almost completely reproduced endothermic peak at the same temperature (at 175˚C). This clearly indicates that the physical constraint to the mesogenic unit in the BH6 is trivial, which in turn suggests the strong physical constraint for the case of the main-chain type liquid-crystalline polymer. Another interesting DSC results are shown in Fig. S5b for the BH6POn, whereby the second-run heating DSC curve appears in the almost identical temperature range for the case of the first-run with a decreased peak area. This result suggests an intermediate extent of the physical constraint to the mesogenic unit because of the main-chain nature of the prepolymer type of the BH6POn.

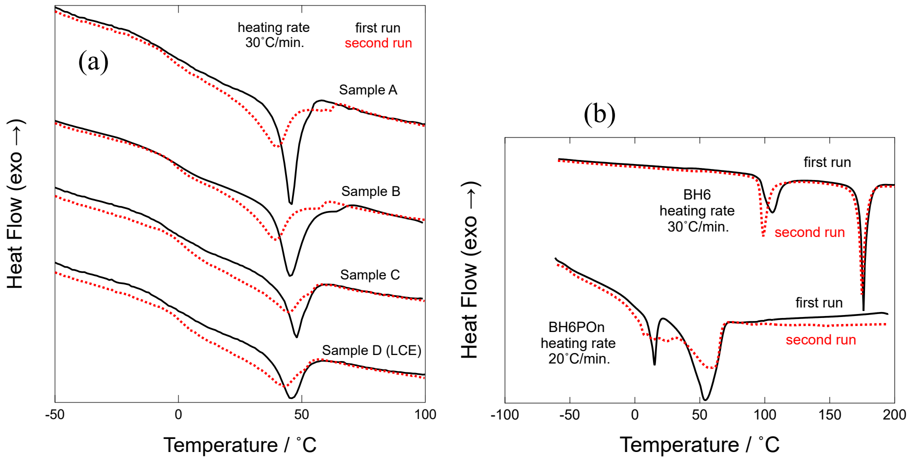

Figure S5. Comparison of the results of the heating DSC curves (with the heating rate of 20 or 30 ˚C/min) for the first run and second run for (a) samples A-D and (b) BH6 and BH6POn.

4. SAXS analysis of the grain size

According to the method we reported in a literature [ref.1] the grain size was estimated in the framework of the Hosemann's paracrystal theory using the width (the integral breadth) of reflection peaks (Δq). After appropriate correction for the peak broadening due to the apparatus (or beamline collimation error), Δq is evaluated from the raw data (the SAXS profile). Then, the Hosemann plot (Figure S6) was made by taking the square of Δq as a function of the fourth-power of m (the order of the peak as m = 1, 2, 3, 4, …). After Hosemann, the following relationship is given.

$$(\Delta q)^2 = \left(\frac{2\pi}{D_G}\right)^2 + \left(\frac{2\pi^3 g^4}{< d >}\right) m^4 \#$$

Here, D_G denotes the grain size (in the direction perpendicular to the reflection planes) and g is the paracrystalline distortion factor given by $g \equiv \Delta d/<d>$ with Δd and $<d>$ being the standard deviation of the distribution of d (the plane spacing) and the mean of d, respectively. The approximated line is drawn in Figure S6 to pass through the first and second points while ignoring the third point. The reason of ignoring the third point is to avoid overestimation of the value of the y intercept of the approximated line in case when three data points are all taken into account. The value of the y intercept even larger than that of the first data point, indicating the lower value of the grain size as compared to the case when the Scherrer's equation is used. As the Hosemann's method is to rule out the contribution of the paracrystalline distortion, the evaluated grain size should be larger than that for the case of using Scherrer' equation. Thus, the third data point was out of consideration for the evaluation. From the y intercept of the approximated line drawn in Figure S6, the value of D_G = 27.2 nm was evaluated.

Ref.1 Ohnogi, H.; Sasaki, S.; Sakurai, S. Evaluation of Grain Size by Small-Angle X-Ray Scattering for a Block Copolymer Film in Which Cylindrical Microdomains Are Perpendicularly Oriented. *Macromolecular Symposia* **2016**, *366* (1), 35–41. https://doi.org/10.1002/masy.201650044.

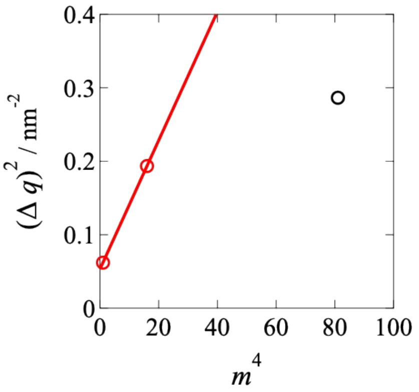

Figure S6. Hosemann plot by taking the square of Δq as a function of the fourth-power of m (the order of the peak as m = 1, 2, 3, 4, …).
